# Nudge to better care - blood cultures and catheter-related bloodstream infections in Germany at two points in time (2006, 2015)

**DOI:** 10.1186/s13756-018-0432-z

**Published:** 2018-11-21

**Authors:** Florian Salm, Frank Schwab, Michael Behnke, Frank M. Brunkhorst, André Scherag, Christine Geffers, Petra Gastmeier

**Affiliations:** 1 Institute for Infection Prevention and Hospital Epidemiology, Medical Center – University of Freiburg, Faculty of Medicine, Freiburg, Germany; 2 Institute of Hygiene and Environmental Medicine, Charité – Universitätsmedizin, Berlin, Germany; 3 National Reference Center for the Surveillance of Nosocomial Infections, Berlin, Germany; 4 Center for Sepsis Control and Care, Jena University Hospital, Jena, Germany; 5 Center for Clinical Studies, Jena University Hospital, Jena, Germany; 6 Department of Anesthesiology and Intensive Care Medicine, Jena University Hospital, Jena, Germany; 7 Institute of Medical Statistics, Computer Sciences and Documentation, Jena University Hospital, Jena, Germany

**Keywords:** Infection control, Bloodstream infections, Sepsis, Blood cultures, Surveillance, Healthcare associated infections

## Abstract

**Background:**

Blood cultures (BCs) are the gold standard for diagnosing sepsis and are prerequisite for a targeted antibiotic treatment and essential for patient outcomes. Aim of the study was to analyze the frequency of BCs, the rate of central line-associated bloodstream infections (CLABSIs) and to study the association between both parameters on intensive care units in Germany over time.

**Methods:**

Cross-sectional studies at two points in time (2006, 2015) on ICUs participating in the German hospital infection surveillance system. CLABSIs were defined according to the Center for Disease Control and Prevention (CDC). Univariable and multivariable analyses were performed using generalized linear models.

**Results:**

A total of 639 ICUs participated in 2006 or 2015 and 90 ICUs (“*core group*”) in both years. Overall, 2,427,921 patient days from 644,575 patients were analyzed. In the ICU core group the frequency of BCs per 1000 patient days doubled from 57.8 (interquartile range [IQR] 29.8–101.2; 2006) to 128.2 (IQR 71.6–183.2; 2015). In the same time, the pooled median CLABSI rate decreased from 0.8 (IQR 0–1.9; 2006) per 1000 central-line catheter days to 0.2 (IQR 0–0.9; 2015).

**Conclusions:**

From 2006 to 2015 the frequency of BCs increased on ICUs in Germany and is now within the recommended 100 to 200 BCs sets per 1000 patient days.

## Background

Early and sufficient diagnostics to identify the causative pathogen is prerequisite for a timely therapy start, a targeted antibiotic treatment and most importantly for the patient outcome. It is essential to become aware of the pathogen and potential antibiotic resistances [1, 2]. Sufficient diagnostic with BC can reduce mortality, length of stay on the intensive care unit and furthermore hospital expense [3, 4]. A targeted antimicrobial therapy can shorten the use of antibiotics and lower the selection pressure and the development of antibiotic resistances, it is one of the core elements of antibiotic stewardship approaches [5]. A range of 100–200 BC sets per 1000 patient days is recommended [6–8], especially because BSI detection rate depends on the frequency of the performance of BCs. Low performers have lower BSI rates compared to wards with a higher frequency in BC diagnostics [8, 9]. Therefore, the frequency of BCs per ward is an important quality indicator if BSI rates are compared. Over the last decade there were a lot of approaches to improve patient safety in critically ill patients by reducing the prevalence of central line-associated bloodstream infections (CLABSIs) [10–13] and by increasing the frequency of performing BCs when there is a suspicion of a BSI [14–16].

The goal of this investigation was to evaluate the frequency of BCs and the influence on the detection rate of CLABSIs and the association between both parameters on ICUs in Germany at two points in the year 2006 and 2015.

## Methods

In Germany, the majority of acute care hospitals participate voluntary in the German hospital infection surveillance system (KISS, “Krankenhaus-Infektions-Surveillance-System”) which is a surveillance system with confidential data feedback [17, 18]. Data were included from ICUs participating in KISS. The surveillance in this module includes invariable different indicator infections like infections of the lower respiratory system (pneumonia and bronchitis), urinary tract infection and (laboratory-confirmed) BSIs. BSIs are defined according to the Center for Disease Control and Prevention (CDC) [19]. A BSI is central catheter-associated (CLABSI) when on the day with the first symptoms of the infection (“infection day”) the patient had a catheter for at least 3 days. It must be mentioned, that the CDC definitions for CLABSI with coagulase-negative staphylococci (CNS) changed. Since 2011, at least two positive BCs with CNS are necessary to meet the criteria of a CNS-BSI. Before 2011, it was sufficient if at least one BC was positive with CNS and the attending physicians started an antimicrobial therapy. Therefore, all CLABSIs with CNS as a pathogen were excluded from the analysis. The CLABSI rate is calculated as the quotient of total BSI of patients with a central-line catheter divided by the total per 1000 central-line days (for more detail please see: nrz-hygiene.de). One BC is defined as a blood collection with no matter how many bottles but at least with one pair of aerobe und anaerobe BC bottles.

The German National Reference Center for Surveillance of Nosocomial Infections provides reference data for each healthcare-associated infection and works with social norm feedback. Mean, median and the 75th percentile are core parameters. Values within the upper quartile are highlighted. For selected infections and particular wards (e.g. neonatal intensive care units) a *Standardized Infection Ratio* (SIR) is provided and is calculated by the number of observed infections divided by the number of expected infections. The SIR is a parameter to help estimate whether the value is higher than in other wards with same characteristics. In addition, the data are graphically processed as a distribution curve and as funnel plots. Outlier values are also highlighted in the graphical representation.

2006 and 2015 a cross-sectional survey among the KISS participants was carried out to collect the blood culture density (BCD). BCD is defined as frequency of BCs per 1000 patient days per ICU. The method of the cross-sectional study conducted in the year 2006, is described in detail elsewhere [9]. Ten years later, in 2015, a cross-sectional study was carried out using the same methods as in the first investigation. Within the participants from 2006 (*n* = 223) and 2015 (*n* = 506), 90 ICUs provided data in both years and will be called *core-group* in the following.

### Statistical analysis

In the descriptive analysis we calculate pooled mean, median and interquartile range (IQR) in total and stratified by year, type of hospital and hospital size (</> 600 beds), type of ICU, the device use, the invasive ventilation rate and length of stay. Differences between categories were tested by Chi-squared test. The rates of the years 2006 and 2015 in the core-group were tested by Wilcoxon test for paired samples and in the entire cohort by Wilcoxon-Mann-Whitney test for unpaired samples.

To investigate the association between the incidences of CLABSI and the frequency of BC univariable and multivariable analysis were performed using generalized linear models (GLM). In the models, we used negative-binomial distribution as probability distribution and the logarithm of central venous catheter (CVC) days was treated as offset parameter. To adjust for confounding the following variables were considered in the regression analysis: type and size of hospital, type and size of ICU, device use of CVC, ventilation and urinary tract catheter and length of stay. In the univariable analyses incidence rate ratios (IRR) were calculated for the individual factors. In the multivariable analyses, adjusted IRRs for BC density categories were calculated and all variables with *p* < 0.1 in the univariable analyses were included following a stepwise backward variable selection (excluding a variable from the model if *p* > 0.05 in the type III test).

All analyses were performed using SPSS [IBM SPSS statistics, version 23, Somer, NY, USA] and SAS 9.4 [SAS Institute, Cary, NC, USA].

### Ethical approval

All data were anonymous and collected in accordance with the German recommendation for good epidemiological practice with respect to data protection [20]. As a federal law, the German Protection against Infection Act (“Infektionsschutzgesetz” §23) regulates the prevention and management of infectious diseases in humans. All hospitals are obliged to continuously collect and analyse healthcare-associated infections and drug-resistant pathogens [21]. These data are reported regularly to the National Reference Centre for the Surveillance of nosocomial infections. Ethical approval and informed consent were therefore not required.

## Results

**Fig. 1:**
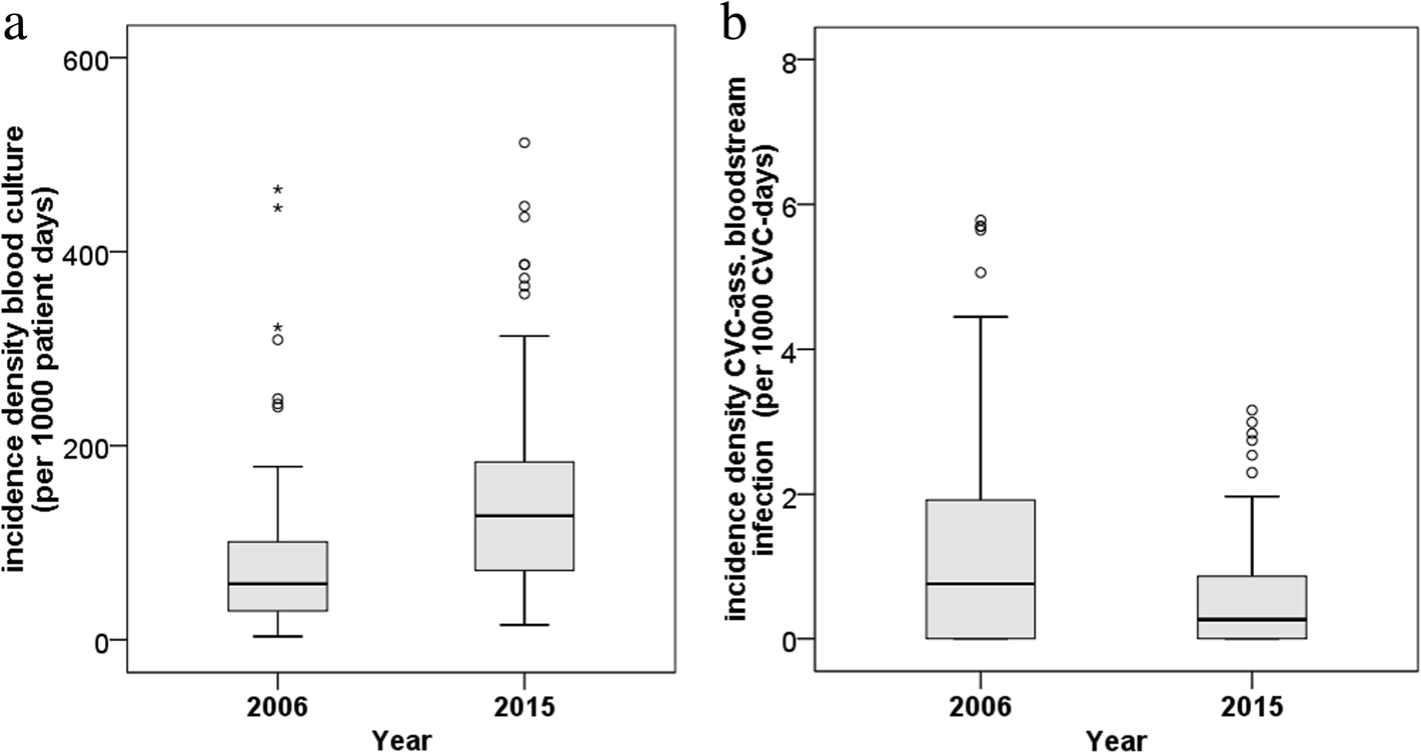
**a** Incidence densitiy of blood cultures in the core group (*n* = 90), **b** Incidence densitiy of central line-associated bloodstream infection in the core group (*n* = 90)

**Fig. 2:**
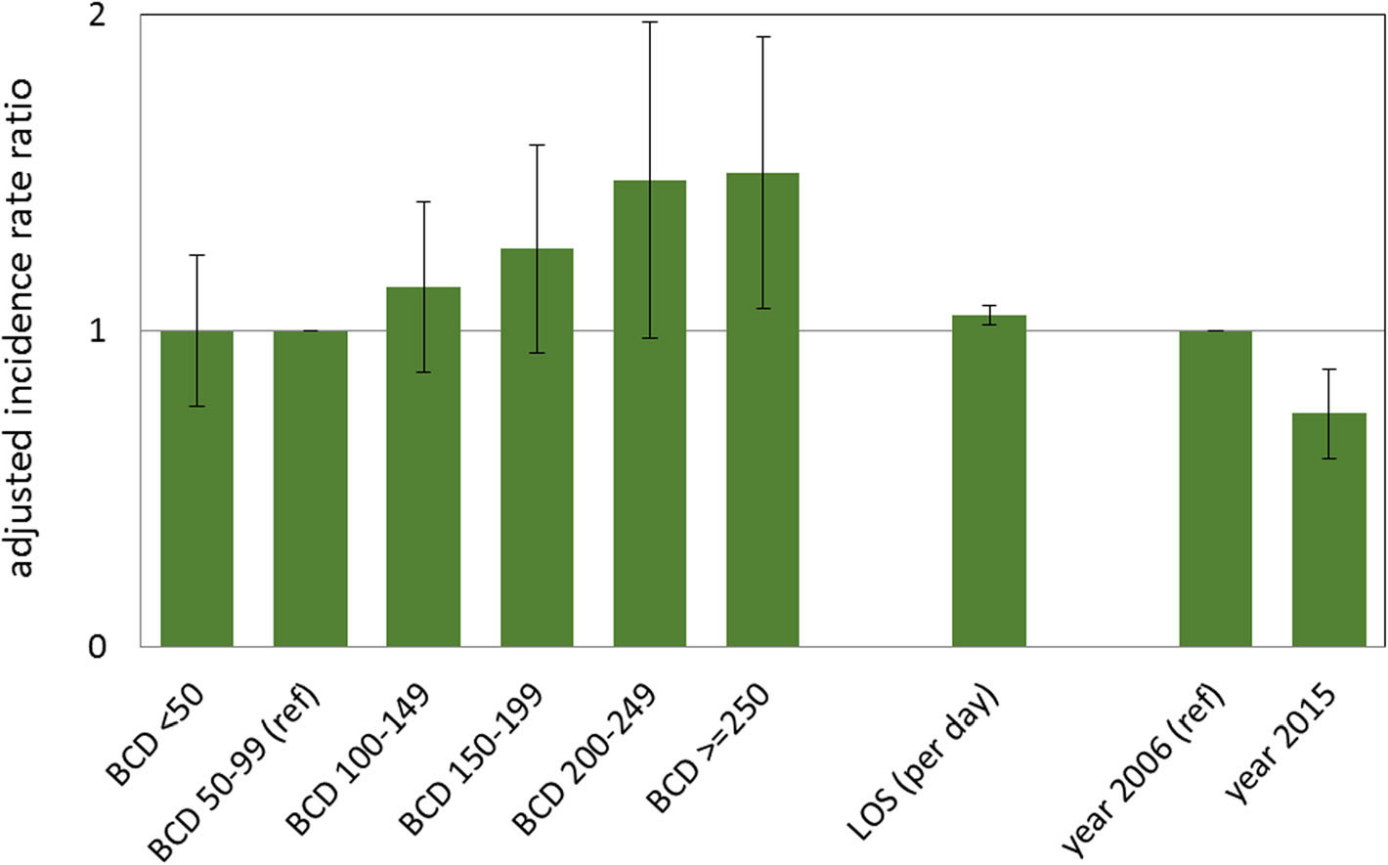
Adjusted incidence rate ratios for blood culture density categories with the outcome central line-associated bloodstream infection (per 1000 CVC days), results of the multivariable regression analysis of 639 wards, 2006 & 2015. Whiskers represent 95% confidence interval. BCD, blood culture density per 1000 patient days; ref., reference in the model; LOS, length of stay

A total of 639 ICUs participated in 2006 or 2015 and 90 ICUs in both years. Overall, 2,427,921 patient days from 644,575 patients were analyzed. Over 1352 CLABSIs with no CNS as pathogen and 343,388 BCs were reported. Wards characteristics can be seen in Table 1. In the ICU core group the BCD doubled from 57.8 (interquartile range [IQR] 29.8–101.2; 2006) to 128.2 (IQR 71.6–183.2; 2015, see Fig. 1a). In the same time, the pooled median CLABSI rate decreased from 0.8 (IQR 0–1.9; 2006) per 1000 central-line catheter days to 0.2 (IQR 0–0.9; 2015, see Table 1 and Fig. 1b). The crude and adjusted incidence rate ratios for the BCD categories compared to 50–99 BC per 1000 patient days can be found in Table 2 and Fig. 2. Wards with 100–199 BC per 1000 patient days did not detect significantly more CLABSIs compared to those wards with 50–99 BC per 1000 patient days. ICUs with more than 250 BCs per 1000 patient days had a 1.50 (95% confidence interval [CI] 1.07–2.11) higher risk to detect CLABSIs compared to ICUs with a low frequency of BCs (50–99- BCs per 1000 patient days). The adjusted IRR for the outcome CLABSI increased with higher frequency of BCs, length of stay and decreased significantly comparing the years 2006 and 2015 (IRR 0.74, 95%CI 0.60–0.92).

**Table 1:** Intensive care units (ICU) characteristics – data from all ICUs (*n* = 639^**^) and from the core group (*n* = 90) from two time points year 2006 and year 2015

Characteristics/ Outcomes	Total ICUs (*n* = 639^**^)		Core Group (*n* = 90)		Core group (*n* = 90)	
	2006 (*n* = 223)	2015 (*n* = 506)	2006 (*n* = 90)	2015 (*n* = 90)	Diff. 2015–2006	*p*-value^*^
Size of ICU						
ICU beds, median (IQR)	11 (8–14)	12 (9–16)	11 (9–14)	11 (9–14)	–	–
ICU > =12 beds, N (%)	100 (44.8)	285 (56.3)	50 (55.6)	50 (55.6)		
Type of ICU, N (%)						
Surgical	40 (17.9)	77 (15.2)	15 (16.7)	15 (16.7)		
Interdisciplinary	108 (48.4)	285 (56.3)	41 (45.6)	41 (45.6)		
Medical	47 (21.1)	73 (14.4)	21 (23.2)	21 (23.2)		
Neurosurgical	4 (1.8)	9 (1.8)	2 (2.2)	2 (2.2)		
Neurological	4 (1.8)	11 (2.2)	2 (2.2)	2 (2.2)		
Pediatric	7 (3.1)	9 (1.8)	3 (3.3)	3 (3.3)		
other^a^	13 (5.8)	42 (8.3)	6 (6.7)	6 (6.7)		
Size of hospital						
Hospital beds, median (IQR	490 (274–841)	431 (270–724)	511 (289–890)	511 (289–890)	–	–
Hospital > = 600 beds, N (%)	85 (38.5)	174 (34.4)	36 (40.0)	36 (40.0)		
Type of hospital						
Tertiary hospital, N (%)	67 (30.0)	130 (25.7)	41 (45.6)	41 (45.6)		
Patients, median (IQR)	741 (506–1036)	814.5 (492–1191)	763.5 (489–1053)	722 (434–1112)	–	–
Patient days, median (IQR)	2744 (1854-3643)	3245 (2183-4365)	2744 (1915-3624)	3028 (2153-4215)	–	–
CLABSI rate^c^, median (IQR)	0.6 (0.0–1.3)	0.5 (0.0–1.2)	0.8 (0–1.9)	0.2 (0–0.9)	− 0.01 (− 1.14–0.02)	0.001
Blood culture density^a^, median (IQR)	60.0 (32.6–107.9)	115.4 (67.6–177.4)	57.8 (29.8–101.2)	128.2 (71.6–183.2)	54.8 (1.6–119.1)	0.001
Length of stay, median (IQR)	3.6 (2.7–5.2)	3.8 (3–5.5)	3.6 (2.8–5.5)	4.2 (2.9–6.2)	0.3 (−0.5–1.3)	0.025
CVC use^b^, median (IQR)	67.3 (49.5–82.1)	66.6 (52.9–80.4)	70.8 (54.2–83.7)	69.0 (57.7–82.7)	0.6 (−6.3–8)	0.672
Invasive ventilation use ^b^, median (IQR)	34.1 (22.7–49.9)	36.8 (26.1–50.4)	35.3 (21.9–52.0)	37.1 (26.5–55.6)	3.1 (− 6.4–10.3)	0.078
Urinary tract catheters use ^b^, median (IQR)	81.3 (70.5–91.5)	85.4 (75.5–92.0)	83.7 (71.6–93.0)	86.7 (78.2–92.4)	2.5 (−2.8–10.0)	0.003

*Abbreviations*: *Diff* difference, *IQR* interquartile range, *CVC* central venous catheter, *CLABSI* central-line-associated bloodstream infection

^*^Wilcoxon-rank-sum test for paired sample; ^**^ 90 ICUs collected data in 2006 and 2015

^a^per 1000 patient days

^b^per 100 patient days

^c^per 1000 CVC-days

**Table 2:** Results from the univariable and multivariable regressions analysis using generalized linear models^a^ with the outcome central line-associated bloodstream infection

Parameter		Univariable Analysis			Multivariable Analysis		
	Category /unit	IRR	95%CI	*p*-value	IRR	95%CI	*p*-value
blood culture density (per 1000pd)	< 50	1.07	0.81–1.41	0.639	1.00	0.76–1.32	0.996
	100–149	1.07	0.81–1.4	0.637	1.14	0.87–1.50	0.343
	150–199	1.18	0.87–1.6	0.283	1.26	0.93–1.71	0.139
	200–249	1.38	0.91–2.09	0.127	1.48	0.98–2.23	0.061
	> = 250	1.46	1.04–2.05	0.029	1.50	1.07–2.11	0.019
	50–99	1 = reference			1 = reference		
Length of stay (days)	per day	1.05	1.02–1.08	0.001	1.05	1.02–1.08	0.001
Year (compared to year 2006)	2015	0.83	0.68–1.02	0.079	0.74	0.60–0.92	0.006
Invasive ventilation use (per 100 pd)	per 1%	1.01	1–1.01	0.004			
CVC use (per 100 pd)	per 1%	1	0.99–1	0.143			
Urinary tract catheter use (per 100 pd)	per 1%	0.99	0.99–1	0.013			
type of ICU	medical	1.49	1.15–1.94	0.002			
	surgical	1.17	0.9–1.51	0.239			
	neurosurgical	1.27	0.62–2.61	0.514			
	neurological	1.26	0.61–2.57	0.534			
	pediatric	1.27	0.6–2.66	0.533			
	other^a^	1.81	1.27–2.58	0.001			
	interdisciplinary	1 = reference					
Size of ICU	> 12 beds	1.11	0.91–1.34	0.306			
type of hospital	tertiary	1.59	1.24–2.03	<.001			
size of hospital	> 600 beds	1.37	1.13–1.65	0.001			

*IRR* incidence rate ratio, *CI* confidence interval, *pd* patient days, *CVC* central venous catheter, *ICU* intensive care unit

^a^The generalized linear models (GLM) with the outcome CLABSI and negative-binomial distribution as probability distribution and the logarithm of CVC days as offset parameter analyzed 729 yearly observations from 639 different ICUs in the years 2006 and 2015

## Discussion

We conducted 2006 and 2015 cross-sectional surveys on, in total, 639 intensive care units in Germany. We found an increase in the frequency of BCs and a significant decrease of catheter-related primary bloodstream infections. An important question is what has caused the strong increase in the BC performance?

In 2007, the German DRG- (Diagnosis Related Groups) reimbursement system changed and for sepsis, a new diagnosis code (R.65) was implemented. It became mandatory to perform at minimum two sets of BCs for the reimbursement of a R.65 patient case [22]. It can be assumed that the change in the DRG-reimbursement system influenced the BCD. Furthermore, several projects addressed this issue over the last years and tried to increase guideline adherence and the implementation of evidence-based diagnostic standard in the care of patients with suspicion of a bloodstream infections [15, 23]. It is well described that higher frequency of BCs are associated with a higher detection rate of BSIs [8, 9]. After a linear trend between culturing rates and the detection of BSIs with higher frequency of BCs a saturation effect can be found, but the optimal frequency of BCs is not well described yet [24]. Focusing on used DRG codes (R.65) for sepsis and sepsis shock, the clinical correlates of BSI, the annual number rose significantly between 2007 to 2013 [25]. In contrast, the CLABSI rate decreased enormously from 2006 to 2015. Potentially, the CLABSI rate decreased due to the different infection and control measures to improve patient safety in critically ill patients by implementing intervention bundles focusing on proper insertion techniques and managing of the CVCs over the last decade [10–13]. In the United States there are approaches to reduce preventable health-care associated infections with *Pay for Performance* policy [26, 27]. Some authors emphasize that reduced payments for CLABSIS did not influence the overall CLABSI rate [27]. On the other hand, from 2008 to 2014 the rate of CLABSIs decreased about 50% in the United States [28], while the BC rate declined from 2008 to 2010 [26]. Calderwood and colleagues highlighted that after implementation of the *Pay for Performance* policy the CLABSI rates decreased in hospitals operating at a financial loss, but in those operating at a financial profit the CLABSI rate was not affected by the policy [29]. In summary, the impact of the *Pay for Perfomance* system on the CLABSI rate in the United States is still unclear.

Nevertheless, considering that in Germany we found a similar decrease in the CLABSI rate without reimbursement penalties, we must critically scrutinize *Pay for Performance* approaches, especially when the outcome can be influenced by diagnostic methods.

However, the burden of health-care associated bloodstream infection is very high with estimated 160.000 cases and a number of about 25.000 attributable deaths in Europe per year [30]. Some investigations pointed out that German inpatient care physicians still have deficits concerning the knowledge of BC sampling; however, the results of this study suggest a positive upward trend in the frequency of BC sampling [31, 32]. Raupach-Rosin et al. reported that the majority of the physicians do not adhere to diagnostic guideline recommendations (two sets of BC from two different sites) [31].

The results are limited by the point that the 1976 reported BSI occurred after the 3rd day according to the surveillance protocol and are only primary BSIs. In contrast, the 343,388 reported BCs are from 1st day until discharge. It must be also considered that the median length of stay on the ICU per patient was less than 4 days. In this investigation, it remains unclear how many BCs were done due to primary and how many due to secondary BSI diagnosis. Nevertheless, from 2006 to 2015 the BCD increased enormously on ICUs and is now within the recommended range for BC of 100 to 200 BCs sets per 1000 patient days [6]. While the large number of observed ICUs (*n* = 639) with over two million patient days under surveillance is a strength of this study, the temporal comparison was limited to the core group of 90 ICUs. However, this core group had consistent ward characteristics over the 10-year period.

## Conclusion

In conclusion, we must assume that many of the BC diagnostics are associated with secondary BSIs. Despite the expected parallel increase of BSI rates a decrease of primary BSI was observed. This underlines our observations of a decreasing trend of BSI rates in ICUs participating in the German hospital infection surveillance system [17].

## Abbreviations

BC: Blood culture
BCD: Blood culture density
BSI: Bloodstream infection
CLABSI: Central line-associated bloodstream infection
CNS: Coagulase-negative
CVC: Central venous catheter
GLM: Generalized linear models
ICU: Intensive care unit
IQR: Interquartile range
IRR: Incidence rate ratios
SIR: Standardized infection ratio
